# Selective and Efficient Arsenic Recovery from Water through Quaternary Amino-Functionalized Silica

**DOI:** 10.3390/polym10060626

**Published:** 2018-06-07

**Authors:** Oscar Valdés, Adolfo Marican, Yaneris Mirabal-Gallardo, Leonardo S. Santos

**Affiliations:** 1 Vicerrectoría de Investigación y Postgrado, Universidad Católica del Maule, 3460000 Talca, Chile; ovaldes@ucm.cl; 2 Instituto de Química de Recursos Naturales, Universidad de Talca, 3460000 Talca, Chile; amarican@utalca.cl; 3 Instituto de Ciencias Químicas Aplicadas, Facultad de Ingeniería en Construcción, Universidad Autónoma de Chile, 3460000 Talca, Chile; yaneris.mirabal@uautonoma.cl; 4 Laboratory of Asymmetric Synthesis, Instituto de Química de Recursos Naturales, Universidad de Talca, 3460000 Talca, Chile

**Keywords:** quaternary ammonium, arsenic, vinyltrimethoxysilane, graft polymerization, silica

## Abstract

The free-radical graft polymerization of acryloxyethyl-trimethylammonium chloride onto commercial silica particles was studied experimentally for extraction of arsenic ions from water. Two steps were used to graft acryloxyethyl-trimethylammonium chloride (Q) onto the surface of nanosilica: anchoring vinyltrimethoxysilane (VTMSO) onto the surface of silica to modify it with double bonds and then grafting Q onto the surface of silica with potassium persulfate as an initiator. The products were characterized by Fourier-transform infrared (FT-IR), the thermogravimetric analysis (TGA), scanning electron microscopy (SEM), ^13^C, ^29^Si nuclear magnetic resonance (NMR), and X-ray powder diffraction (XRD). The results showed that it is easy to graft Q onto the surface of silica under radical polimerization. The morphology analysis of silica and modified silica indicated that the silica decreased the size scale after modification. Q/VTMSO-SiO_2_ was tested for its ability to remove arsenic from drinking water. The results show that the new silica hybrid particles efficiently remove all arsenate ions. In addition, Q/VTMSO-SiO_2_ showed better sorption capacities for other metal ions (such as copper, zinc, chromium, uranium, vanadium, and lead) than a commercial water filter.

## 1. Introduction

Arsenic contamination of tap water is reported in many undeveloped countries and is considered a primary environmental health hazard throughout the world. Arsenic is an element found in the Earth’s crust and is mobilized through natural processes. As a consequence of these anthropogenic activities, arsenic enters the water cycle [1]. However, agricultural and industrial practices such as mining activities, combustion of fossil fuels, use of arsenic pesticides, electronics production waste, crop desiccants, runoff from glass production, and use of arsenic additives to livestock feed can lead to an increase in the concentration of arsenic in water sources [1]. For these reasons, the greatest risk of human contamination by arsenic is probably due to the ingestion of polluted water.

It is known that small doses of arsenic in the human body cause or aggravate many diseases such as cardiovascular, pulmonary, immunological, neurological, peripheral vascular, and endocrine and different types of cancer (e.g., lung, bladder, skin, prostate, kidney, etc.) [2,3]. With this in mind, the World Health Organization suggests 0.05 ppm as the maximum level of arsenic in drinking water [4]. This element is naturally found in water in two oxidation states, arsenite (AsO_3_^3−^) and arsenate (AsO_4_^3−^). The presence of each oxidation state depends on the redox potential, pH condition, other ions, and microbial activity. Arsenate species are less toxic than arsenite, which is more widespread in aerobic surface waters. On the other hand, trivalent arsenites predominate in moderately reduced anaerobic environments such as groundwater [5]. Many countries like the USA, China, Chile, Bangladesh, Argentina, Poland, Mexico, Taiwan, Canada, Hungary, New Zealand, Japan, and India have recently reported cases of chronic exposure to high amounts of arsenic in water [1]. Therefore, arsenic in natural water is a worldwide dilemma that has not yet been resolved.

Several techniques have been developed to eliminate arsenic from aqueous solutions, including adsorption, oxidation, precipitation, electrocoagulation, membrane permeation, and biological methods, with far less attention paid to adsorption [6]. Low-cost adsorbents can be used for arsenic removal from drinking water and groundwater with high efficiency. Adsorption involves the use of granular materials for the selective elimination of arsenic from water with or without pH adjustment and spent media regeneration. Therefore, many adsorptive media have been used, for example activated alumina, ion exchange resin, elemental iron or iron compounds, organic polymers, chars, coal, red mud, blast furnace slag (BFS), kaolin clay, and silica sand, among others. Iron hydroxide or zero-valent irons are the most widely used [1]. There are many variables to select a proper sorbent media to remove arsenic from drinking water, such as: the range of arsenic concentrations, other elements and their concentrations, adsorbent dose, filtration of treated water, adjustment of pH, post-treatment difficulties, handling of waste and, finally, proper operation and maintenance. The other contaminants found in tap water that could be occupying the active sites of the adsorbent are generating competition for the retention of the target pollutant, reducing treatment run times. For these reasons, adsorbent selection is a complex decision.

Sorbent technologies, which are successful in the laboratory, may fail in field conditions [1]. Additionally, these techniques generate mostly used adsorbents or collected residues polluted by arsenic species, and they must be properly treated as hazardous wastes. Therefore, the selection of the correct sorbent technologies for efficient arsenic removal from contaminated water is an essential issue regarding environmental remediation and human health.

Water-soluble polymers are a new type of purification technique for arsenic. Water-soluble polymers undergo interactions with solvent and other high- and low-molecular-weight species present in aqueous solutions [7,8]. There are different cationic polymers containing an exchanger quaternary ammonium group to remove arsenic species from aqueous media. These polymers have the following advantages: soluble in water with a high number of functional groups, exchange anions with arsenic species, and, thus, have higher efficiency. These polymers can be used with ultrafiltration membranes [9,10]. The problem with this technique is that these technologies need the combination of electrochemical oxidation and adsorption or ultrafiltration procedures, and therefore are not suitable for industrial applications.

To address this inconvenience and leverage the benefits previously mentioned, we propose a new organic-inorganic hybrid material design using a vinyltrimethoxysilane-acryloxyethyl-trimethylammonium chloride polymer (Q/VTMSO-SiO_2_) and ion-exchange properties for removing arsenate anions from water.

Finally, it is important to note that organic-inorganic hybrid materials have attracted significant attention [11,12,13,14,15] because of their potential applications in many surface-based technologies such as composite materials, biomaterials, adhesion and wetting, molecular recognition, microfluidics, chemical sensing, and organic synthesis [16,17,18,19,20,21,22]. In addition, this kind of material has excellent properties, such as mechanical properties, biodegradation and abrasion resistance, thermal stability, flame retardance, and gas barrier properties [23,24,25,26].

Presently, there are many approaches for modifying inorganic surfaces with polymers but the most frequently used is covalent attachment [27,28]. Specifically, three methods give the best results: the “grafting to” method [29,30], where the end-functionalized polymers react with an appropriate surface; the “grafting from” method [31,32], where polymer chains are grown from initiator-terminated self-assembled monolayers, and, finally, the “grafting onto” method [33], where surface copolymerization is through a covalently linked monomer.

In this article we report on the second type of approaches to covalently graft polymer chains on the surface of inorganic particles described previously. In particular, we prepared acrylic-functionalized silica particles and their copolymerization with acrylic monomer using radical polymerization for making an acrylic-silica hybrid composite. In addition, we focus on the chemical and structural characterization and the morphology. Moreover, the use of the acrylic monomer with a quaternary ammonium group significantly enhanced the ion-exchange properties of arsenate anions, as mentioned earlier.

## 2. Experimental Section

### 2.1. Materials

All chemical products were analytical grade. Silica (SiO_2_) (Fluka, Santiago, Chile) with a particle size of 0.063–0.020 mm (70–230 mesh) and a mean pore size of 60 Å was used as support. Hydrochloric acid (Fluka) and Vinyltrimethoxysilane (VTMSO, Sigma-Aldrich Chemical, Santiago, Chile) were used without purification. Toluene (Merck, Santiago, Chile) was distilled and dried with metallic sodium, which was then used as the solvent for the silylation process. Potassium persulfate (K_2_S_2_O_8_, Fluka) was employed as the initiator for acryloxyethyl-trimethylammonium chloride (Q, Sigma-Aldrich Chemical) free-radical polymerization. Pure Milli-Q water (Elga Labwater, High Wycombe, UK) was employed during the arsenic recovery experiments. The commercial polymer was obtained from a water filter purchased in a local market. We used normal drinking water for the metal affinity experiments between the filler from the purchased water filter and Q/VTMSO-SiO_2_.

### 2.2. Silylation

Initially, the silica particles were cleaned with 1% HCl solution for 1 h at room temperature, followed by several rinses with water until it had a neutral pH to clean the surface. After that, particles were filtered and dried at 110 °C in a vacuum oven overnight to remove excess surface water. Then, the hydroxylated particles were immersed in 7 mM toluene solution of VTMSO at 30 °C under stirring for 18 h to form VTMSO-silylated particles. Subsequently, the silylated particles were washed with a large volume of toluene and methanol to remove the excess of VTMSO and dried at 80 °C until a constant weight. The VTMSO-silylated particles were obtained as a white powder and the quantity of grafted VTMSO on the surface of the silica particles was calculated by TGA (TA Instruments, New Castle, DE, USA) using the following equation [34]:

(1) Grafting density (molm2)=(W1100−W1) × 100− WSiMSSpec × 100×106,

where *W*_1_ is the weight loss from 110 to 700 °C, corresponding to the decomposition of the VTMSO; *M* (g/mol) is the molar mass of the degradable part of the grafted molecule (105 g/mol); and *S*_spec_ (314 m^2^/g) and *W*_Si_ are the specific surface area and the weight loss of silica determined before grafting, respectively. The *S*_spec_ were determined by nitrogen sorption isotherms with a standard BET method using a Tristar 3020 Micrometrics apparatus (Micromeritics, Norcross, GA, USA). Prior to the measurements, the silica particles were heated at 140 °C for 24 h under a vacuum.

### 2.3. Graft Polymerization

**Scheme 1:**
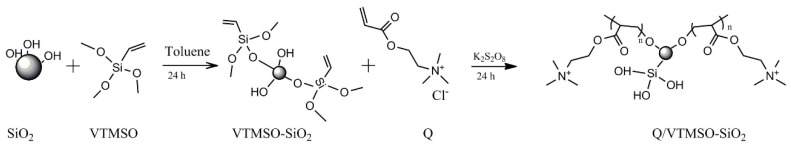
Radical grafting procedures on silica with Q investigated in this work.

Free-radical graft polymerization of acryloxyethyl-trimethylammonium chloride (Q) onto VTMSO-silylated particles was carried out in a necked flask using 0.01 M of K_2_S_2_O_8_ as initiator (Scheme 1). The initial monomer concentration (Q) was 80% of VTMSO-silylated particles weight using a predetermined volume of water. The reaction was performed under a nitrogen atmosphere at 60 °C, using magnetic stirring overnight. After the reaction time, the reaction mixture was filtered and washed with water to eliminate the excess of unreactive Q. Finally, the acryloxyethyl-trimethylammonium chloride on VTMSO-silylated particles (Q/VTMSO-SiO_2_) was dried under a vacuum until constant weight was attained. The grafting percentage of modified silica was determined by thermogravimetric analyses. The percentage of acryloxyethyl-trimethylammonium chloride based on VTMSO-silylated particles was calculated by the following equation [35]:

(2) $$Polymer\;grafting\;\left(\%\right)=\frac{A}{B}\times 100,$$

where *A* is the weight of organic compounds grafted onto the VTMSO-silylated particles (mg), obtained from the TGA data; and *B* is the weight of VTMSO-silylated particles (mg).

### 2.4. Characterization

Fourier-transform infrared (FT-IR) spectrophotometer (Nicolet Nexus 470, Nicolet Instruments, Offenbach, Germany) was employed to obtain spectra of acryloxyethyl-trimethylammonium chloride on VTMSO-silylated particles (Q/VTMSO-SiO_2_). All spectrums were obtained in KBr pellets from an average of 32 scans with 4 cm^−1^ resolution within the 4000–400 cm^−1^ spectral interval. The Q/VTMSO-SiO_2_ was characterized by a comparison of the FT-IR spectra of the silica and VTMSO-silylated particles. Thermogravimetric analyses of the thermally treated samples were performed with a TA Instruments Q500 thermal gravimetric analyzer. The sample powders were heated in a nitrogen flow speed of 50 mL/min from ambient temperature to 700 °C at a rate of 10 °C/min. Carbon-13 and Silicon-29 Nuclear Magnetic Resonance spectra for solid samples were obtained on a Varian INOVA 300 spectrometer (Varian, Inc., Palo Alto, CA, USA) equipped with a Cross Polarization-Magic Angle Spinning (CP/MAS) solid-state probe operating at resonance frequency of 74.5 MHz and 5 KHz for ^13^C and ^29^Si, respectively. X-ray diffraction (XRD) was recorded on a Rigaku RIX 3100 spectrometer (Rigaku Corp., Tokyo, Japan) with copper anode as the source of X-rays at λ = 1.54 Å. The morphologies of SiO_2_, VTMSO-SiO_2_ and Q/VTMSO-SiO_2_ were observed by a scanning electron microscope (JEOL JSM 5610LV, Tokyo, Japan) using a 20 kV accelerating voltage.

### 2.5. Metal Affinity Studies

Solid Phase Extraction (SPE) cartridges were made with 5 mL polyethylene disposable syringes, using silanized glass wool as support for the polymers. 500 mg of polymer was used per experiment. These filters were mounted on a Manifold Visiprep SPE vacuum system (Bellefonte, PA, USA) to achieve constant and reproducible flows. In the first stage, 1 L of arsenic model solution (0.1 mg·L^−1^) was freshly prepared using Milli-Q water. Six aliquots of 160 mL of this solution were passed through the cartridge containing Q/VTMSO-SiO_2_ to determine its affinity by arsenic. In the second stage, arsenic spiked drinking water was used to evaluate the affinity of Q/VTMSO-SiO_2_ and commercial polymer in parallel. In this way, the retention capacity of the polymers by As was evaluated using an experimental design, considering the elution volume (100, 300, and 500 mL) through the filters, and the concentration of drinking water (16.38, 87.42, and 409.2 µg·L^−1^). The values of these experimental variables were coded between –1 and 1 so that they had the same statistical weight. Finally, the determination of arsenic and other metals present in the drinking water, such as copper, zinc, chromium, uranium, vanadium and lead (Cu, Zn, Cr, U, V, and Pb) was carried using ICP-MS iCAP Q equipment (ThermoFisher Scientific, Bremen, Germany). The parameters used in the ICP-MS equipment were air flow 13.50 L/min, acetylene flow 2.00 L/min, burner height 13.50 mm, slit width 0.2–1.0 nm, and lamp current 4–10 mA.

## 3. Results and Discussion

### 3.1. ^13^C and ^29^Si NMR Analysis

**Figure 1:**
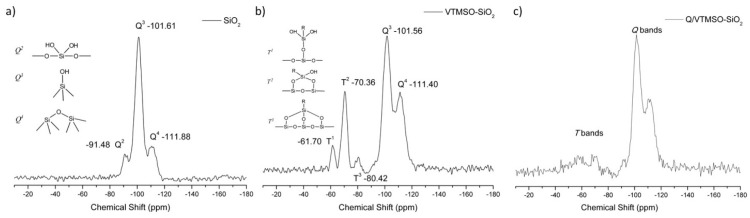
Solid-state ^29^Si NMR spectra of SiO_2_ (**a**), VTMSO-SiO_2_ (**b**), and Q/VTMSO-SiO_2_ (**c**). Inset: possible structure details with signal assignment.

Radical grafting from polymerization of Q on VTMSO-SiO_2_ particles requires three working steps: (a) silica activation; (b) functionalization of silica with vinyltrimethoxysilane to form silica–C=C (VTMSO-SiO_2_); and (c) functionalization of VTMSO-SiO_2_ with Q to form silica–N^+^(CH_3_)_3_ (Q/VTMSO-SiO_2_). Figure 1 shows the ^29^Si NMR spectra of a typical Q/VTMSO-SiO_2_ sample and the VTMSO-SiO_2_ and silica precursors.

The ^29^SiNMR spectrum of silica gel for chromatography is shown in Figure 1a and shows typical peaks at −11.88, −101.61, and −91.48 ppm, which can be identified with silicon sites in a reasonable surface model. These signals are assigned to the silicon atoms bound to four other oxygens called Q^4^ moieties at the silica gel surface, to the surface silicon atoms attached to three oxygens (Q^3^) moieties and one OH group, and finally the −91.48 ppm resonance is assigned to surface silicon atoms bonded to two oxygen and two hydroxyl groups, respectively. It is important to note that these values are in reasonable qualitative agreement with the values found by ^29^Si CP/MAS for silica reported by Kedong et al. [36]. Figure 1b shows the VTMSO-SiO_2_ spectra; the signals corresponding to Q^3^ and Q^4^ decrease in contrast to the Q^2^ shift, which virtually disappears, thus verifying the silylation of silica gel. Meanwhile, three new peaks, T^3^, T^2^, and T^1^, are generated, indicating that the VTMSO was triply, doubly, and singly bonded to silica particles. Finally, we obtained the spectra of Q/VTMSO-SiO_2_ (Figure 1c). The remarkable feature in this spectrum is the decrease in intensity of the region where the T sites appear, thus corroborating the successful radical grafting from polymerization of Q on silica.

**Figure 2:**
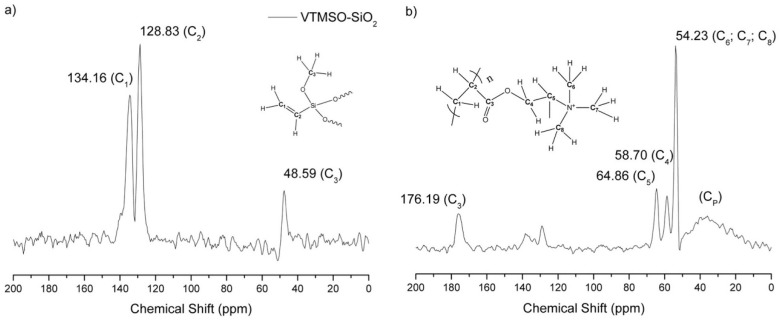
Solid-state ^13^C NMR spectrum of VTMSO-SiO_2_ (**a**) and Q/VTMSO-SiO_2_ (**b**). Inset: structure details with signal assignment.

Figure 2 shows the solid state ^13^C NMR spectra of VTMSO-SiO_2_ and Q/VTMSO-SiO_2_. The VTMSO-SiO_2_ spectra is characterized by three signals corresponding to the carbons that form double bonds and a methyl group founded in 134.16, 128.83, and 48.59 ppm, respectively. The Q molecule has one carbonyl, two methylene bridges, three methyl groups, and one double bond, and its molecular structure attached to the functionalized silica gel has been verified based on a comparison of the solid state ^13^C NMR spectra of Q/VTMSO-SiO_2_ and VTMSO-SiO_2_. The Q/VTMSO-SiO_2_ spectra is characterized by the almost disappearance of the ethenic double bond signs as a result of the radical grafting from polymerization of Q on silica. The presence of vinylics signs is probably a consequence of double bonds belonging to a VTMSO-SiO_2_ that is not spatially available for polymerization. Furthermore, the generation of a new sign centered in approximately 40 ppm corresponds to the CH_2_ groups of the skeleton of the polymeric chain and the other signals corresponding to the molecular structure of Q, corroborating the presence of Q on VTMSO-SiO_2_. The Q/VTMSO-SiO_2_ spectra data agree with the results reported by Oscar et al. [37]. However, it was difficult to be certain from these spectra how many Q and VTMS molecules reacted with silica gel. We solved this problem using the thermogravimetry technique and the results will be discussed later.

### 3.2. FT-IR Analysis

**Figure 3:**
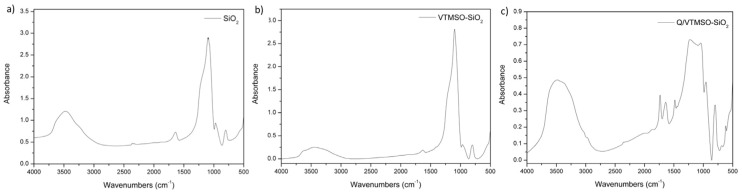
FT-IR spectrum of SiO_2_ (**a**), VTMSO-SiO_2_ (**b**), and Q/VTMSO-SiO_2_ (**c**).

Therefore, the processing FT-IR spectroscopy was performed for SiO_2_, VTMSO-SiO_2_ and Q/VTMSO-SiO_2_ to examine their chemical structures. These spectra are compared in Figure 3a–c, respectively. In all samples there were strong characteristic absorption peaks that were assigned to the silane carbon bonds, Si–CH at 800 cm^−1^ and absorption peaks at around the 1100 cm^−1^ region characteristic of the methoxy groups Si–OCH_3_ and any siloxane cross-links (Si–O–Si) that were formed during the silylation of silica by VTMSO. Also, we observed the absorption bands of OH stretching at 3432 cm^−1^ and H–O–H bending at 1634 cm^−1^characteristic of silica. It is important to note that in the FT-IR spectra of VTMSO-SiO_2_ the signal corresponding to the vinyl group overlapped due to the intensity of the other silica signals.

However, comparing the SiO_2_ and VTMSO-SiO_2_ spectra, the changes of intensities, shape, and position of the silica signals in the 1100–800 cm^−1^ region indicated that silane grafting reactions had occurred. Finally, the Q/VTMSO-SiO_2_ spectra show characteristic absorption bands corresponding to the principal functional groups present in the Q molecule and VTMSO-SiO_2_. Thus, the spectrum showed a broad structureless band at 3493 cm^−1^ because of the OH groups, two peaks at 1734 and 1481 cm^−1^, which were assigned to a C=O and N^+^(CH_3_)_3_ groups, respectively, and one sharp peak at 1198 cm^−1^, which is for the C–O group. At 956 cm^−1^ there appears as a singular band presented in the Q monomer spectrum, which may be related to the C–O stretching vibration in a –O– fragment [38]. Bands at 2965 cm^−1^ (CH stretching) and 1500–1300 cm^−1^ (various C–H bending) were also detected. All these indicate that the synthesized particles have combined chemical structures of silica, VTMSO, and Q, corroborating the results obtained by ^29^Si and ^13^C NMR.

### 3.3. TGA Analysis

**Figure 4:**
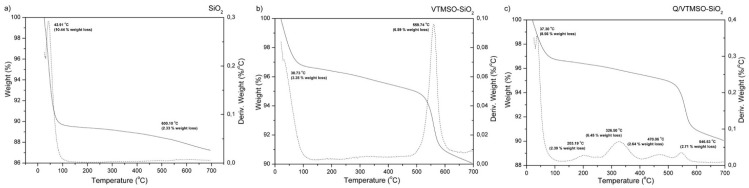
TG-DTG thermograms of SiO_2_ (**a**), VTMSO-SiO_2_ (**b**), and Q/VTMSO-SiO_2_ (**c**).

The thermogravimetric analysis (TGA and derivate) of SiO_2_, VTMSO-SiO_2_, and Q/VTMSO-SiO_2_ are shown in Figure 4a–c, respectively. These studies resulted in some interesting information about the thermal stability and fraction weight loss of prepared compounds. Figure 4a shows that the thermogram obtained for SiO_2_ had a first loss of 14.90% until 62 °C due to physically adsorbed methanol, and a second loss in the range of 110–800 °C corresponding to the condensation of surface silanol groups. As for VTMSO-SiO_2_, the high temperature required to decompose and evaporate the organic content of the silanized silica demonstrated that the silane coupling agent (VTMSO) was strongly bound to the particle surface and suggested a covalent bond (see Figure 4b). This decomposition event starting at 500 °C corresponded to the decomposition temperature of VTSMO covalently attached to silica particles, and the weight loss was 6.59%. Calculations based on TGA showed that the grafting density of grafted vinyl group is 0.235 mmol/m^2^ according to Equation (1).

The thermogram corresponding to the decomposition of Q/VTMSO-SiO_2_ showed quite a different mechanism (see Figure 4c) due to the poly(Q) presence in the silanized silica. Its decomposition occurred in five steps starting at 120, 256, 408, and 516 °C, respectively. The total weight loss was about 23% of the Q/VTMSO-SiO_2_ degradable part. The degradation mechanism consisted in random chain scissions and the ammonium salt decomposition in the case of poly(Q), the polymer grafting was 14.36%. On the other hand, it could be that the presence of a VTMSO degradation signal at high temperature was due to the vinyl group that is not available spatially for polymerization. The obtained poly(Q) degradation data agreed with the results reported by Oscar et al. [38]. Finally, it is important to note that the slight weight loss, about 6%, of three thermograms observed at around 40 °C was likely caused by the methanol used in washing. These results reinforced the data obtained by FT-IR, ^13^C, and ^29^Si NMR.

### 3.4. XRD Analysis

**Figure 5:**
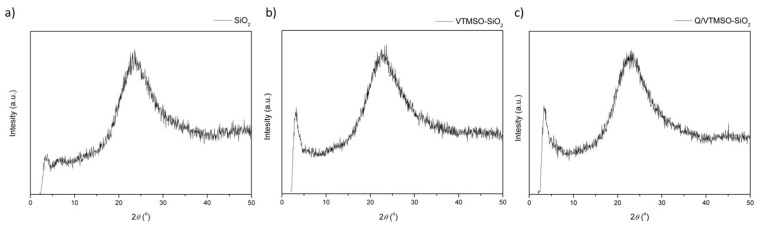
XRD patterns of SiO_2_ (**a**), VTMSO-SiO_2_ (**b**), and Q/VTMSO-SiO_2_ (**c**).

The XRD analysis of SiO_2_, VTMSO-SiO_2_, and Q/VTMSO-SiO_2_ showed that the amorphous condition of the silica was unaffected by the silylation and grafting processes (See Figure 5). Thus, we observe no apparent change in XRD patterns. The XRD pattern of three materials displayed two intense peaks in the 2θ range of 0.9–2.5, which is associated to the thin monolayer of trimethylsilyl moiety and the amorphous diffraction peak of silica-gel. In general, after silylation and grafting processes, the first peak increased the intensity due to the VTMSO presence. In conclusion, the XRD patterns suggested that surface treatment of silica had no evident effect on its crystal structure.

### 3.5. SEM Analysis

**Figure 6:**
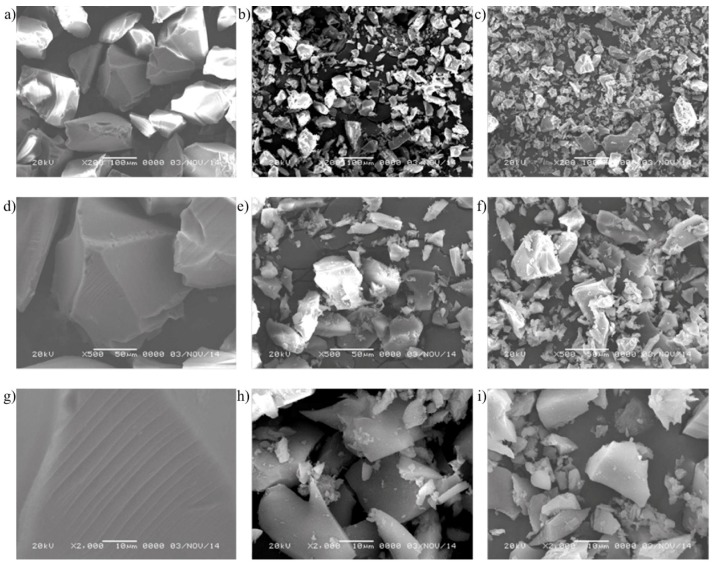
SEM images of SiO_2_ (**a**,**d**,**g**), VTMSO-SiO_2_ (**b**,**e**,**h**), and Q/VTMSO-SiO_2_ (**c**,**f**,**i**) with different magnifications.

Morphologies of the silica particles before/after were observed to examine the difference in morphologies aroused by the VTMSO coating and Q grafting, respectively. Figure 6 shows the SEM images of SiO_2_, VTMSO-SiO_2_, and Q/VTMSO-SiO_2_ with different magnifications. The silica particles looked like a crystal had an irregular cubic shape and a polydispersed size. In addition, the silica particles had a smooth and clean surface. After modification by VTMSO and Q, the particle surface became rougher and decreased in size, but they maintained their irregular shape (see Figure 6b). It is important to note that the addition of Q over silanized silica particles decreased in size even more. This is because the surface of Q/VTMSO-SiO_2_ contained a large number of quaternary ammonium groups that did not interact and the OH groups of silica decreased dramatically. Finally, the particle surface was covered with nanoscale lumps, which were believed to be the VTMSO and poly(Q) coating in the case of VTMSO-SiO_2_ and Q/VTMSO-SiO_2_, respectively.

### 3.6. Arsenic Retention Properties of Q/VTMSO-SiO_2_

**Figure 7:**
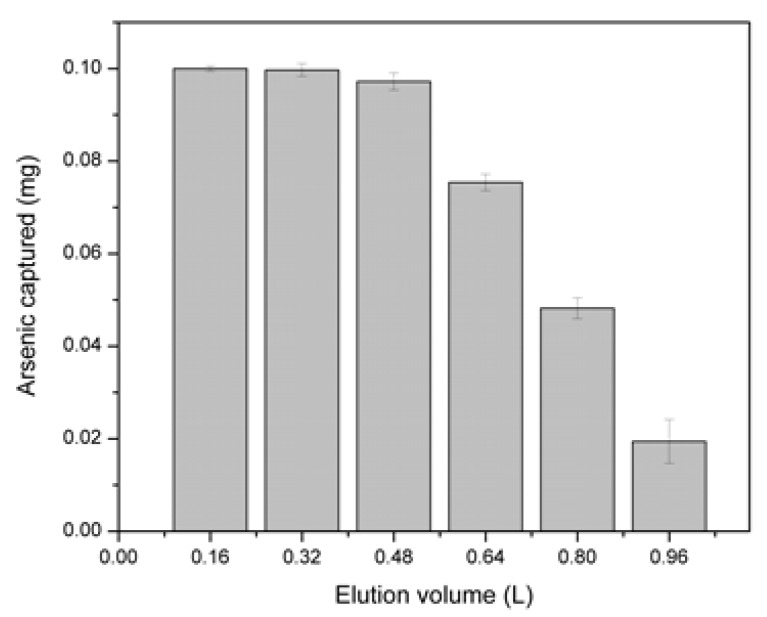
Affinity assays between Q/VTMSO-SiO_2_ and arsenic solution (0.1 mg·L^−1^).

In Figure 7, the results of affinity assays between Q/VTMSO-SiO_2_ and model arsenic solution are shown. In elutions 1 and 2, the cartridge was able to capture all the arsenic present in the model solution. In the third elution, the capture capacity of the cartridge decreased to 97.7%. The fourth elution showed a capture capacity of 74.9%. In the fifth elution, the cartridge capture capacities fell to 48.2%. Finally, in the sixth elution, the cartridge capture capacities were only 19%.

Considering these results, 1 g of Q/VTMSO-SiO_2_ could treat up to 320 mL of 0.1 mg·mL^−1^ arsenic solution, capturing the total amount of arsenic. If we want to decrease the concentration of arsenic to a half value, we can treat up to 800 mL of 0.1 mg·mL^−1^ arsenic solution. Scaling these results, if we use a filter filled with 1 kg of Q/VTMSO-SiO_2_, we can treat 320 L of water with arsenic concentration of 0.1 mg·mL^−1^, capturing 100% of the present arsenic. If we want to get to the international limits of arsenic in drinkable water (0.05 mg·mL^−1^), we can treat up to 800 L.

### 3.7. Metal Affinities of Q/VTMSO-SiO_2_ Fill and Commercial Water Filter Fill

We compared the capacity of removing metal contaminants in drinking water using Q/VTMSO-SiO_2_ fill and a fill obtained from commercial water filters acquired from a commercial store. Table 1 present the results of the experimental design used to evaluate the affinity of fill from commercial water filters.

**Figure 8:**
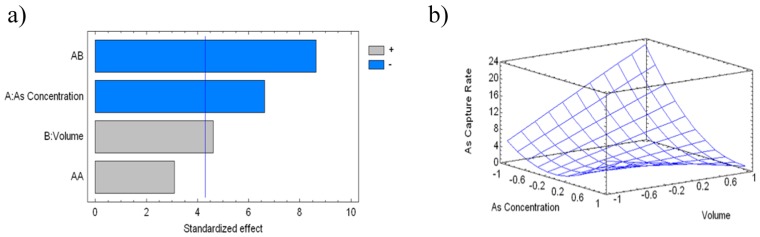
(**a**) Standardized Pareto chart for As capture rate, due to commercial polymer treatment (where: A, As concentration; B, Eluted volume; and AB, AA and BB interactions. Blue line represents critical *t*-value, 95% confidence); and (**b**) estimated response surface.

The results obtained using the experimental design are shown through a Pareto chart. In Figure 8, the Pareto chart and estimated response surface for the As capture experiments are presented using the fill from commercial water filters.

Considering the statistical significance of the variables, the equation of the model is:

$$As\;Capture\;Rate=3.55-4.90\times A+2.87\times B-6.00\times A\times B\;\left(R^{2}=37.28\right).$$

According to the model, the optimum As capture predicted by this filter fill was 23.06%. The optimum experimental conditions were a volume of 500 mL and a concentration of 16.38 ppb. In this way, this filter fill would allow the treatment of 500 mL of drinking water contaminated with As, reducing its concentration from 16.38 to 12.6 ppb of As.

Following the same procedure, we tested Q/VTMSO-SiO_2_ under the optimum experimental conditions, measuring the affinity for metals naturally present in drinking water. Table 2 presents the results of the experimental design used to evaluate the affinity of Q/VTMSO-SiO_2_.

**Figure 9:**
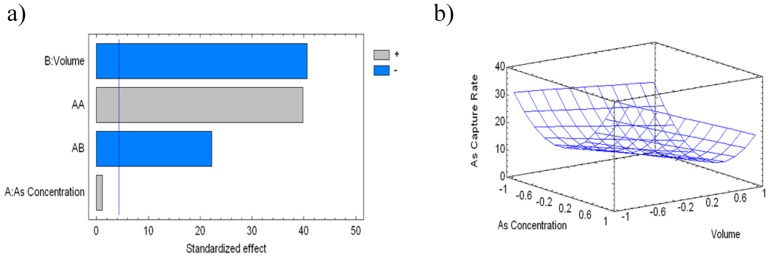
(**a**) Standardized Pareto chart for As capture rate, due to Q/VTMSO-SiO_2_ filler treatment (where: A, As concentration; B, Eluted volume; and AB, AA and BB interactions. Blue line represents critical *t*-value, 95% confidence); and (**b**) estimated response surface.

Based on the results obtained, Figure 9 shows the Pareto chart and the estimated response surface for the As capture experiments using Q/VTMSO-SiO_2_ in drinking water.

According to the statistical significance of the variables, the equation of the model is:

As Capture Rate=10.2−6.36×B+18.6×A^2−3.90×A×B(R2=65.57).

The optimum As capture predicted for Q/VTMSO-SiO_2_ fill was 39.24%. The optimum experimental conditions were a volume of 100 mL and a concentration of 409.2 ppb. In this way, the use of this filter fill would allow the treatment of 100 mL of drinking water contaminated with As, reducing its concentration from 409.2 to 248.6 ppb of As. In addition, 500 mL of As-contaminated water can be treated at 16.38 ppb, decreasing its concentration to 11.33 ppb. The differences observed between the model solution using MilliQ water and drinking water enriched with As are due to the presence of other metals in solution, generating competition for the retention of other metals present.

**Figure 10:**
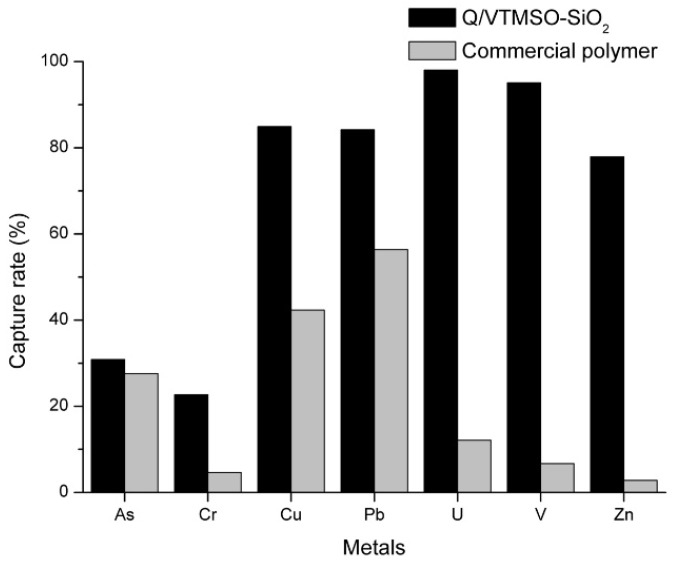
Capture rate of some heavy metals present in drinking water by the commercial polymer and Q/VTMSO-SiO_2_.

Finally, considering the experimental conditions in which the commercial polymer presented a better performance, the experimental conditions of the second experiment (Table 1 and Table 2) were selected and the affinity of both polymers to other metals present in the drinking water was determined. The results are shown in Figure 10. It is observed that the commercial polymer has a lower affinity than Q/VTMSO-SiO_2_ for all the metals present in the drinking water used.

## 4. Conclusions

In summary, new silica hybrid particles were made available by a two-step synthesis. In the first step, silica particles are functionalized with vinyltrimethoxysilane; secondly, they are radically copolymerized with Q. The product was fully characterized and tested for its ability to remove arsenic and other metals present. Remediation of arsenate anions was successfully achieved using Q/VTMSO-SiO_2._ The results showed that 1 g of Q/VTMSO-SiO_2_ could treat up to 320 mL of 0.1 mg·mL^−1^ arsenic solution, capturing the total amount of arsenic from drinking water. In addition, Q/VTMSO-SiO_2_ showed better sorption capacities for other metal ions such as copper, zinc, chromium, uranium, vanadium, and lead than the fill from a commercial water filter. These results suggest that the strategy of using modified silica with quaternary ammonium groups onto surfaces could lead to new devices for drinking water purification.

## Figures and Tables

**Table 1:** Experiments for affinity by As performed using fill from commercial water filters.

Experiment	As concentration [μg·L^−1^]	Volume [mL]	As captured [μg·L^−1^]	As capture rate [%]
1	16.38 (−)	100 (−1)	0.130	0.790
2	16.38 (−1)	500 (1)	4.52	27.6
3	87.42 (−0.64)	100 (−1)	3.24	3.71
4	87.42 (−0.64)	500 (1)	5.27	6.03
5	409.2 (1)	100 (−1)	26.7	6.52
6	409.2 (1)	500 (1)	9.20	2.25
7	87.42 (−0.64)	300 (0)	8.80	10.1
8	87.42 (−0.64)	300 (0)	10.9	12.5
9	87.42 (−0.64)	300 (0)	11.2	12.8

Numbers between parentheses indicate the entry sequenced dataset for statistical analysis.

**Table 2:** Experiments for affinity by As, performed using Q/VTMSO-SiO_2_.

Experiment	As concentration [μg·L^−1^]	Volume [mL]	As captured [μg·L^−1^]	As capture rate [%]
1	16.38 (−1.00)	100 (−1)	4.31	26.30
2	16.38 (−1.00)	500 (1)	5.05	30.80
3	87.42 (−0.64)	100 (−1)	25.20	28.80
4	87.42 (−0.64)	500 (1)	8.33	9.530
5	409.2 (1.00)	100 (−1)	156.00	38.20
6	409.2 (1.00)	500 (1)	80.90	19.80
7	87.42 (−0.64)	300 (0)	14.90	17.00
8	87.42 (−0.64)	300 (0)	14.20	16.30
9	87.42 (−0.64)	300 (0)	14.50	16.50

Numbers between parentheses indicate entry sequenced data set for statistical analysis.
